# Effect of different dietary protein levels and amino acids supplementation patterns on growth performance, carcass characteristics and nitrogen excretion in growing-finishing pigs

**DOI:** 10.1186/s40104-019-0381-2

**Published:** 2019-09-16

**Authors:** Yumei Zhao, Gang Tian, Daiwen Chen, Ping Zheng, Jie Yu, Jun He, Xiangbing Mao, Zhiqing Huang, Yuheng Luo, Junqiu Luo, Bing Yu

**Affiliations:** Key Laboratory of Animal Disease-Resistance Nutrition, Animal Nutrition Institute, Sichuan Agricultural University, 46# Xinkang Road, Yucheng District, Yaan, 625014 Sichuan China

**Keywords:** Growing-finishing pigs, Growth performance, Isoleucine, Low protein diets, Nitrogen excretion, Valine

## Abstract

**Background:**

This study was conducted to determine the effects of different dietary protein levels and amino acids supplementation patterns in low protein diets on the growth performance, carcass characteristics and nitrogen excretion in growing-finishing pigs.

Forty-two barrows (25.00 ± 0.39 kg) were randomly assigned to 7 diets. Diet 1: the high crude protein diet with balanced for 10 essential amino acids (EAAs). Diet 2: the medium crude protein diet with 2% (approx) decreased protein level of Diet 1 and balanced 10 EAAs. Diet 3: the low crude protein diet with 4% decreased protein level of Diet 1 and balanced 10 EAAs. The protein levels of Diet 4, 5, 6 and 7 were the same as that of Diet 3. Diet 4 was only balanced for lysine (Lys), methionine (Met), threonine (Thr) and tryptophan (Trp); Diet 5 and 6 were further supplemented with extra isoleucine (Ile) or valine (Val), respectively; Diet 7 was further supplemented with extra Ile + Val.

**Results:**

Over the 112 days trial, the reduction of dietary protein by 2% or 4% with balanced10 EAAs significantly decreased nitrogen excretion (*P* <  0.05), but had no effects on growth performance and carcass characteristics (*P* > 0.05). In low protein diet, Val supplementation significantly increased body weight gain at 25–50 kg phase (*P* <  0.05), while Ile supplementation at 75–100 kg phase and 100–125 kg phase significantly reduced the ratio of feed to gain (*P* <  0.05). No effect of different dietary protein levels and amino acids supplementation patterns in low protein diets on carcass characteristics was observed (*P* > 0.05). The total N excretion of pigs supplemented with only Lys, Met, Thr and Trp was numerically higher than that of pigs fed with extra Ile, or Val, or Ile + Val diets.

**Conclusion:**

In low protein diet, Val is more required than Ile in the early growing phage (25–50 kg), while Ile becomes more required in the late growing and finishing phage (75–125 kg).

**Electronic supplementary material:**

The online version of this article (10.1186/s40104-019-0381-2) contains supplementary material, which is available to authorized users.

## Background

On the economic and environmental perspectives, the reduction of dietary crude protein and the supplementation with crystalline amino acids were the effective strategies for the swine industry to reduce the cost and pollution [1, 2]. It has been reported that the total N excretion is reduced by approximately 8% for every 1 % unit reduction in dietary CP [3]. Previous studies showed that the 4% reduction of CP in diet does not influence growth performance in pigs from growing to finishing when supplemented with crystalline Lys, Trp, Thr and Met [4–7]. However, the opposite result was reported thatreducing dietary protein by 4% and supplementing Lys, Trp, Thr and Met decrease the average daily gain (ADG) in 20–50 kg pigs [8]. Moreover, reducing dietary protein levels by 5% in 20–50 kg pigs, significantly decreases growth performance [9], probably due to the deficiency of other essential amino acids (EAAs) in the low protein diets.

Ten EAAs were required in pigs for the maximum of nitrogen deposition [10]. However, it was impossible to balance 10 EAAs in diets due to the high cost. Thus, supplementation of the first four limiting amino acids (Lys, Met,Trp and Thr) or even fewer is a common strategy in pig production. As mentioned, when the dietary protein level was reduced by more than 4%, the supplementation of the only first four limiting amino acids might affect the growth performance in growing-finishing pigs [8, 9, 11]. Thus, it is necessary to find an economical supplementation pattern that can ensurethe normal growth of pigs. Val and Ile are the fifth and sixth limiting AA in growing-finishing pigs [12]. Val, Ile and leucine (Leu) are branched-chain amino acids and play important roles in protein deposition and normal physiological functions in the body. Due to the similar structure, branched-chain amino acids compete with each other for the absorption, transportation and degradation [13]. In diets with corn as the main ingredient, Leu content generally exceeds the needs of pigs, which may decrease the decomposition of Val and Ile and exacerbate the lack of these two amino acids [14, 15]. Several studies have investigated the effects of Val and Ile in low protein diets on the growth performance of piglets and growing pigs [12, 16–18]. However, there are limited data available in finishing pigs.

The objective of this experiment was to estimate the effects of different dietary protein levels and amino acids supplementation patterns (including Val and Ile) in low protein diets on growth performance, carcass characteristic and nitrogen excretion of the growing-finishing pigs.

## Methods

### Experimental animals and diets

The experiment was conducted in the Metabolism Laboratory of the Institute of Animal Nutrition, Sichuan Agricultural University (Yaan, China).A total of 42 barrows [(Landrace×Yorkshire) × Duroc; initial body weight 25.00 ± 0.39 kg] were randomly allotted to seven diets based on the initial weight (*n* = 6). The trial lasted 112 days and was divided into four weight phases 25–50 kg, 50–75 kg, 75–100 kg and 100–125 kg. The 7 diets were: Diet 1, the high crude protein diet (HCP), protein levels in the four phases were 17.5%, 16.0%, 14.5% and 13.0%, respectively, and balanced for 10 EAAs; Diet 2, the medium crude protein diet (MCP), based on the NRC (2012) recommendation, protein levels in the four phases were 15.70%, 13.50%, 12.13% and 10.44%, respectively, and 10 EAAs were balanced. The dietary protein level of Diet 3, 4, 5, 6 and 7 (the low crude protein diets, LCP) was decreased by 4% on the basis of Diet 1. Additionally, Diet 3 balanced for 10 EAAs; Diet 4 only balanced for Lys, Met, Thr and Trp; Diet 5 and 6 were further supplemented with extra Ile or Val, respectively; Diet 7 was further supplemented with extra Ile + Val. The NE level of Diet 1 and Diet 2 was 10.36 MJ/kg according to NRC (2012) [19]; the NE level of Diet 3, 4, 5, 6 and 7 was 10.06 MJ/kg according to the results of Yi et al. [8], which found that the NE level should be reduced to obtain optimal carcass characteristics in low protein diet. All pigs were individually housed in stainless-steel metabolism crates (1.8 m × 0.8 m × 1.6 m).

Experimental diets were mainly composed of corn, wheat bran, soybean meal and rapeseed meal. Dietary protein level was reduced by replacing part of soybean meal with corn and wheat bran. Experimental diets were formulated on the basis of the standardized ileal digestible amino acids and NE system. Feed ingredients samples were collected for crude protein and amino acid analysis before the feed formulation, and the standard ileal digestible amino acid of the diets was calculated according to the standard ileal digestibility in the Table of China Feed Composition and Nutritional Value (25th ed., 2014). Nutrients in the diets met or exceeded the nutrient requirement recommended by NRC (2012) except dietary CP and NE (Table 1 and Additional file 1: Table S1-S4). Limitation order of EAAs in low protein diets showed in Table 2.

**Table 1:** Nutrient levels of diets

Weight phase	Nutrient	HCP^3^	MCP^4^ (− 2%)	LCP^5^ (− 4%)
25–50, kg	CP^1^, %	17.42	15.76	13.54
	NE^2^, MJ/kg	10.36	10.36	10.06
50–75, kg	CP, %	16.01	13.70	12.24
	NE, MJ/kg	10.36	10.36	10.06
75–100, kg	CP, %	14.63	12.40	10.70
	NE, MJ/kg	10.36	10.36	10.06
100–125, kg	CP, %	13.11	10.67	9.33
	NE, MJ/kg	10.36	10.36	10.06

CP^1^: Analytical value

NE^2^: According to the prediction equation of Noblet et al. [20] NE/(kcal/kg DM) = (0.700 × DE) + (1.61 × EE) + (0.48 × Starch)-(0.91 × CP)-(0.87 × ADF)

HCP^3^: High crude protein

MCP^4^: Medium crude protein

LCP^5^: Low crude protein

**Table 2:** Limitation order of essential amino acids in low protein diets

Item	25–50, kg Phase	50–75, kg Phase	75–100, kg Phase	100–125, kg Phase
First limiting AA	Lys (54.74, %)	Lys (51.57, %)	Lys (46.67, %)	Lys (44.49, %)
Second limiting AA	Met (64.77, %)	Thr (64.80, %)	Thr (61.26, %)	Thr (60.70, %)
Third limiting AA	Thr (66.52, %)	Trp (68.14, %)	Trp (64.29, %)	Trp (62.56, %)
Fourth limiting AA	Trp (71.04, %)	Met (68.21, %)	Met (69.56, %)	Ile (72.29, %)
Fifth limiting AA	Val (78.76, %)	Ile (76.16, %)	Ile (76.17, %)	Met (73.81, %)
Sixth limiting AA	Ile (79.27, %)	Val (81.07, %)	Val (80.73, %)	Val (83.20, %)
Seventh limiting AA	His (92.59, %)	His (96.95, %)	Phe (96.39, %)	
Eighth limiting AA	Phe (96.49, %)	Phe (97.38, %)	His (98.97, %)	

The percentages in parentheses represent the ratio of the amino acids content in the low protein diet(−4%) without addition of synthetic amino acids) to the NRC (2012) recommendation

Pigs were fed at 8:00, 14:00 and 20:00 *ad libitum* with free access to water. Pigs were individually weighed at each weight phase shift, and the daily feed consumption of each pig was recorded. The data was used to calculate average daily feed intake (ADFI), average daily gain (ADG) and feed-to-gain ratio (F /G ratio).

### Nitrogen balance andammonia emissionstudy

In the last four days of each phase, all pigs were subjected to a 4-day total faeces and urine collection. At the end of the collection period, four day’s fecal samples from each pig were pooled. 500 g fecal sample was dried in a forced-draft oven at 65 °C, grounded through a 0.45-mm sifter, and kept at − 20 °C for further analysis. Urine was collected and recorded daily at the same time as the faecal collection. At the end of the collection period, four day’s urine samples from each pig were pooled, and a 100 mL subsample stored at − 20 °C for further analysis. Additionally, on the last day of the collection, fresh feces (100 g) and urine (100 mL) from each pig were immediately placed into a 10-L bucket. The ammonia concentration in the bucket at 0 h and 4 h were measured by portable ammonia detector (APES-NH3(200)-H, Empaer, Shenzhen, China).

### Blood sample collection

At the end of the trial (day 113), blood samples were collected via *jugular vein* puncture after a 12-h overnight fasting. Blood samples were centrifuged (3,000×*g* for 15 min at 4 °C) and serum samples were stored at − 20 °C.

### Assessment of carcass characteristics and organ index

After blood sampling, all pigs were slaughtered following a standard procedure at the end of the trial. After exsanguination and evisceration, carcass was split through the midline, and the hot carcass weight (including kidney and leaf fat) was recorded to calculate dressing percentages. Internal organs were removed and weighed to calculate organ index, which is the percentage of the organ weight to the live weight of the pig. The other carcass traits were measured (obtained from the left side of the carcass), including average backfat thicknesses (average of first- rib, last-rib and last-lumbar fat thickness), carcass length and loin-eye area (The measurement position of loin-eye area is the cross-section of the longissimus dorsi muscle at the junction of the thoracolumbar segment, and the measurement method is used a vernier caliper to measure the maximum height and width of the eye muscle, and the calculation formula is: loin-eye area = length × width × 0.7).

### Laboratory analysis of samples

For determining nitrogen-balance, crude protein of experimental diets, faeces and urine samples were analyzed according to AOAC 984. 13 [21].

The concentration of serum urea nitrogen (SUN), total protein (TP) and albumin (ALB) were analyzed using assay kits according to the manufacturer’s instructions (Nanjing Jiancheng Bioengineering Institute, China).

Ammonia nitrogen (NH_3_-N) concentration in serum and faeces was determined spectrophotometrically according to Nessler reagent with yellow coloring and photometering at a wavelength of 420 nm.

All measurements were determined in triplicate.

### Statistical analysis

Diet 1, diet 2 and diet 3 groups were compared to explore the effect of different dietary protein levels in the same amino acid supplementation pattern, diet 3, diet 4, diet 5, diet 6 and diet 7 groups were compared to explore the effect of different amino acid supplementation patterns at the same dietary protein level. All data were analyzed by one-way ANOVA for a randomized complete block design using the SPSS statistical software package (SPSS 20.0).Statistical differences between diets were separated by Duncan’s multiple range tests. All data were expressed as the mean ± SE. A difference was considered significant at *P* <  0.05 and 0.05 < *P* <  0.10 was considered a tendency.

## Results & discussion

The reduced level of protein in the diet requires the increased supplementation of amino acids to maintain the similar growth performance [22]. The balance of different amino acids in diet also affected the utilization of nitrogen and other nutrients. It was reported that 4% lower protein diet with supplementation of the first four limiting amino acids (Lys, Met, Trp and Thr) has no effect on the growth performance of pigs, compared to normal protein diet [4–6]. However, the reduction of more than 4% of protein in diets hinders the growth of pigs, even if supplemented with those limiting amino acids [8, 9, 11]. This may be due to the imbalance of essential amino acids in the low protein diets. The effects of different amino acids supplementation patterns in low protein diets on finishing pigs are still unclear. Therefore, in this study, we estimated the effects of different dietary protein levels and amino acids supplementation patterns (including Val and Ile) in low protein diets on growth performance, carcass characteristic and nitrogen excretion of the growing-finishing pigs.

### Growth performance of growing-finishing pigs

The results of growth performance were shown in Table 3. Reduced dietary protein levels by 2% had no significant effect on growth performance in pigs (*P* > 0.05), which was consistent with previous research [23–25]. We also found that a 4% reduction in dietary protein with balanced for 10 EAAs had no negative effect on pig growth performance (*P* > 0.05). However, if only balanced for Lys, Met, Thr and Trp, 4% reduction of dietary protein increased F/G ratio, indicating other essential amino acids were lacking in the low protein diets. In 25–50 kg pigs, Val supplementation significantly increased ADFI and ADG (*P* <  0.05), while the supplementation of Ile even reduced ADFI. The similar results were observed in Figueroa et al. [16] that the addition of Val or Val + Ile in 5% lower protein diet improves the growth performance in 20 kg growing pigs, compared to control diet. However, the addition of Ile alone significantly decreased ADFI and ADG. The authors suggested that when dietary protein level decreased by 5%, the fifth limiting amino acid was Val instead of Ile. The addition of extra Ile to low protein diets exacerbates deficiency of Val. Val deficiency leads to decreases in feed intake and growth [26]. Moreover, deficiency of Val increases both central somatostatin expression and ghrelin resistance resulting in reduced feed intake [27, 28]. Val activates hypothalamus mTORC1 and GACC, increases the expressions of NPY and MC4R, and thus promotes food intake [29]. In this study, there was no significant difference in growth performance between different amino acids balanced patterns in 50–75 kg phase (*P* > 0.05).The reduction of feed intake in Ile alone group in 50–75 kg phase was smaller than that in 25–50 kg phase, which indicated that the lack of Val was relieved in 50–75 kg phase. In both 75–100 kg phase and 100–125 kg phase, supplementation of Ile alone significantly improved the ADFI and ADG (*P* <  0.05) and decreased F/G (*P* <  0.05), while supplementation of Val even decreased the ADG and increased F/G. It indicated that Ile was more required than Val in 75–100 kg and 100–125 kg phase pigs. The results of growth performance were consistent with the limiting order of the essential amino acid in the low protein diets showed in Table 2, i.e., Val was the fifth limiting amino acid for the growth performance in the 25–50 kg phase, and Ile was the fifth limiting amino acid in the later phase and even rose to the fourth limiting amino acid in the 100–125 kg phase in the present experimental diets.

**Table 3:** Growth performance of growing-finishing pigs fed low protein diet supplemented with different amino acids (*n* = 6)

Item	HCP	MCP (−2, %)	LCP(− 4, %)					*P*_1_	*P*_2_
	10 EAAs	10 EAAs	10 EAAs	Lys, Met,Thr, Trp	Lys,Met,Thr, Trp, Ile	Lys,Met,Thr, Trp, Val	Lys, Met,Thr, Trp, Ile, Val		
**Phase I (25–50, kg)**									
Initial weight, kg	25.4 ± 0.7	25.5 ± 0.8	25.4 ± 0.5	25.5 ± 0.6	25.4 ± 0.7	25.4 ± 0.5	25.8 ± 0.8	0.94	0.98
Final weight, kg	51.5 ± 0.7	51.8 ± 0.6	50.5 ± 2.0^abc^	47.4 ± 2.0^c^	48.3 ± 1.1^bc^	52.0 ± 0.9^ab^	52.3 ± 0.6^a^	0.56	0.05
ADFI, g	1641 ± 55	1607 ± 60	1573 ± 43^bc^	1550 ± 112^bc^	1461 ± 86^c^	1795 ± 56^a^	1715 ± 22^ab^	0.68	0.02
ADG, g	791 ± 33	808 ± 29	761 ± 18^ab^	665 ± 44^b^	691 ± 44^b^	801 ± 26^a^	813 ± 13^a^	0.49	< 0.01
F/G	2.09 ± 0.07	1.99 ± 0.05	2.07 ± 0.02^b^	2.33 ± 0.06^a^	2.12 ± 0.06^b^	2.23 ± 0.08^ab^	2.11 ± 0.04^b^	0.37	0.02
**Phase II (50–75, kg)**									
Initial weight, kg	51.5 ± 0.7	51.8 ± 0.6	50.5 ± 2.0^abc^	47.4 ± 2.0^c^	48.3 ± 1.1^bc^	52.0 ± 0.9^ab^	52.3 ± 0.6^a^	0.56	0.05
Final weight, kg	75.8 ± 1.8	77.0 ± 1.9	76.0 ± 1.5	77.0 ± 2.3	71.7 ± 2.7	76.8 ± 1.6	76.0 ± 1.2	0.92	0.10
ADFI, g	2132 ± 46	2076 ± 146	2351 ± 77	2280 ± 63	2155 ± 106	2306 ± 96	2307 ± 62	0.15	0.53
ADG, g	866 ± 32	844 ± 56	908 ± 43	842 ± 24	838 ± 31	883 ± 34	860 ± 28	0.58	0.51
F/G	2.48 ± 0.08	2.47 ± 0.08	2.63 ± 0.18	2.71 ± 0.05	2.57 ± 0.06	2.62 ± 0.13	2.69 ± 0.05	0.61	0.90
**Phase III (75–100, kg)**									
Initial weight, kg	75.8 ± 1.8	77.0 ± 1.9	76.0 ± 1.5	77.0 ± 2.3	71.7 ± 2.7	76.8 ± 1.6	76.0 ± 1.2	0.92	0.10
Final weight, kg	103.2 ± 2.6	103.9 ± 2.6	104.1 ± 2.1	99.4 ± 2.7	101.7 ± 2.6	102.4 ± 2.9	104.0 ± 1.6	0.60	0.60
ADFI, g	2734 ± 142	2699 ± 143	2703 ± 67	2949 ± 104	2703 ± 140	2709 ± 101	2753 ± 93	0.97	0.41
ADG, g	932 ± 50	902 ± 36	938 ± 34^ab^	947 ± 43^ab^	1000 ± 18^a^	856 ± 52^b^	914 ± 22^ab^	0.80	0.06
F/G	2.94 ± 0.07	2.99 ± 0.13	2.90 ± 0.11^ab^	3.13 ± 0.08^a^	2.71 ± 0.15^b^	3.20 ± 0.16^a^	3.01 ± 0.11^ab^	0.81	0.07
**Phase IV(100–125, kg)**									
Initial weight, kg	103.2 ± 2.6	103.9 ± 2.6	104.1 ± 2.1	99.4 ± 2.7	101.7 ± 2.6	102.4 ± 2.9	104.0 ± 1.6	0.60	0.60
Final weight, kg	123.9 ± 3.2	123.6 ± 3.3	122.8 ± 3.2	118.8 ± 3.4	123.2 ± 3.1	122.3 ± 3.1	120.7 ± 2.1	0.97	0.87
ADFI, g	3080 ± 145	2866 ± 117	3049 ± 135	3146 ± 109	3083 ± 105	3104 ± 103	2829 ± 210	0.52	0.54
ADG, g	958 ± 62	937 ± 68	889 ± 57^ab^	926 ± 52^ab^	1022 ± 47^a^	946 ± 53^ab^	798 ± 38 ^b^	0.95	0.08
F/G	3.25 ± 0.16	3.1 ± 0.21	3.28 ± 0.08^ab^	3.42 ± 0.10^a^	3.03 ± 0.10^b^	3.31 ± 0.13^ab^	3.53 ± 0.14^a^	0.70	0.04
**Overall (25–125, kg)**									
Initial weight, kg	25.4 ± 0.7	25.5 ± 0.8	25.4 ± 0.5	25.5 ± 0.6	25.4 ± 0.7	25.4 ± 0.5	25.8 ± 0.8	0.94	0.98
Final weight, kg	123.9 ± 3.2	123.6 ± 3.3	122.8 ± 3.2	118.8 ± 3.4	123.2 ± 3.1	122.3 ± 3.1	120.7 ± 2.1	0.97	0.87
ADFI, g	2326 ± 77	2270 ± 108	2347 ± 53	2406 ± 74	2271 ± 95	2413 ± 46	2351 ± 76	0.91	0.43
ADG, g	879 ± 22	876 ± 37	877 ± 25	833 ± 29	872 ± 21	865 ± 28	856 ± 11	0.99	0.72
F/G	2.64 ± 0.05	2.60 ± 0.10	2.63 ± 0.04^bc^	2.88 ± 0.04^a^	2.58 ± 0.07^c^	2.80 ± 0.08^ab^	2.75 ± 0.08^ab^	0.89	0.01

*P*_1_ represented the *P* value of ANOVA among the HCP diet, MCPdiet and LCP diet with balancing 10 EAAs

*P*_2_ represented the *P* value of ANOVA among different amino acid balanced patterns of the LCP diets groups

^a,b,c^Superscripts indicated differences among different amino acid balanced patterns of LCP diets groups

### Carcass characteristics and organs index in finishing pigs

In addition to the controversy in the growth performance, the impact of low protein diets on carcass characteristics had also been questioned by many researchers. Kerr et al. found that reduced dietary protein levels and supplemented with synthetic amino acids increases carcass fat deposition, compared to normal protein diet [4]. The increase in carcass fat was due to the enhancement of both net energy density and energy efficiency when reducing the dietary protein level [30, 31]. Both Knowles et al. [32] and Dourmad et al. [33] found that the formulation of low-protein diets with using NE system could reduce the deposition of carcass fat, compared with using DE system. Yi et al. [8] found that reduce NE level in low protein diets improves carcass characteristics which were similar to those of the normal protein diet group. In order to avoid the interference of dietary energy on carcass characteristics, the net energy level of low protein diets (4% reduction) was established based on the result of Yi et al. [8]. No significant effects of dietary protein levels and amino acid balance patterns on carcass characteristics were observed (Table 4). The similar result was also reported in Jiao et al. [34]. Thus, different amino acids supplementation in low protein diets had no significant effects on carcass characteristics.

**Table 4:** Carcass characteristics and organ index of finishing pigs fed low protein diet supplemented with different amino acids (*n* = 6)

Item	HCP	MCP (− 2, %)	LCP(− 4, %)					*P*_1_	*P*_2_
	10 EAAs	10 EAAs	10 EAAs	Lys, Met, Thr, Trp	Lys, Met, Thr, Trp, Ile	Lys, Met, Thr, Trp, Val	Lys, Met, Thr, Trp, Ile, Val		
Carcass weight, kg	87.60 ± 2.54	89.65 ± 2.35	90.74 ± 2.47	88.39 ± 2.31	88.61 ± 2.40	89.03 ± 3.03	86.71 ± 2.34	0.66	0.91
Dressing percentage, %	71.79 ± 1.03	73.77 ± 1.21	74.21 ± 0.47	74.44 ± 0.53	73.04 ± 0.28	72.72 ± 0.85	73.01 ± 0.95	0.21	0.29
Carcass length, cm	113.67 ± 2.36	116.20 ± 3.01	113.43 ± 2.21	112.00 ± 2.19	111.17 ± 1.62	114.33 ± 1.62	112.40 ± 2.27	0.71	0.4
Backfat thickness, cm	2.22 ± 0.17	2.14 ± 0.35	2.13 ± 0.17	2.61 ± 0.14	2.36 ± 0.14	2.43 ± 0.17	2.42 ± 0.17	0.93	0.64
Loin muscle area, cm^2^	57.18 ± 1.62	57.90 ± 6.86	56.92 ± 2.70	50.14 ± 2.10	52.97 ± 2.30	59.68 ± 5.07	58.87 ± 3.30	0.98	0.08
Heart, %	0.32 ± 0.01	0.32 ± 0.01	0.32 ± 0.01	0.34 ± 0.01	0.33 ± 0.01	0.34 ± 0.01	0.32 ± 0.01	0.98	0.63
Liver, %	1.49 ± 0.05	1.31 ± 0.11	1.29 ± 0.05	1.31 ± 0.05	1.30 ± 0.02	1.32 ± 0.05	1.29 ± 0.09	0.13	0.99
Spleen, %	0.12 ± 0.01	0.13 ± 0.00	0.11 ± 0.01	0.12 ± 0.01	0.11 ± 0.01	0.14 ± 0.01	0.12 ± 0.01	0.09	0.21
Lung, %	0.62 ± 0.05	0.57 ± 0.09	0.48 ± 0.05	0.56 ± 0.07	0.63 ± 0.05	0.47 ± 0.03	0.60 ± 0.09	0.31	0.29
Kidney, %	0.28 ± 0.01^x^	0.26 ± 0.00^xy^	0.23 ± 0.01^y^	0.25 ± 0.01	0.23 ± 0.01	0.26 ± 0.01	0.25 ± 0.01	0.05	0.75
Pancreas, %	0.11 ± 0.00^x^	0.09 ± 0.01^y^	0.07 ± 0.00^z^	0.09 ± 0.01	0.10 ± 0.01	0.09 ± 0.01	0.10 ± 0.01	< 0.01	0.20
Thymus, %	0.11 ± 0.01	0.12 ± 0.01	0.10 ± 0.01	0.10 ± 0.01	0.10 ± 0.01	0.09 ± 0.01	0.08 ± 0.01	0.41	0.69
Leaf fat, %	1.32 ± 0.10	1.64 ± 0.19	1.36 ± 0.18	1.71 ± 0.14	1.72 ± 0.05	1.71 ± 0.12	1.35 ± 0.17	0.34	0.13

The organ index represented the ratio of organ mass to pig live weight.

*P*_1_ represented the *P* value of ANOVA among the HCP diet, MCP diet and LCP diet with balancing 10 EAAs.

*P*_2_ represented the *P* value of ANOVA among different amino acid balanced patterns of the LCP diets groups.

^x,y,z^Superscripts indicate differences among different levels of dietary protein

Pancreas and kidney play important roles in the process of protein digestion and metabolism. Pancreatin secreted by the pancreas can degrade proteins into peptide and amino acids. The kidney is an important excretory organ for nitrogen metabolism. It has been reported that reduce dietary protein from 16% to 12% significantly reduces the organ index of the pancreas in 30–60 kg pigs [7]. Peng et al. [35] also found that reduce dietary protein level from 20% to 15.3% significantly decreases the organ index of the liver and pancreas in piglets. The further reduction of dietary protein level from 16.7% to 13.2% significantly decreases the organ index of the kidney. In the present study, reduction of dietary protein levels by 2% or 4%with balancing 10 EAAs decreased pancreas index (*P* <  0.05) (Table 4). Meanwhile, dietary protein levels decreased by 4% also significantly reduced the kidney index (*P* <  0.05). When the dietary protein level was reduced, the metabolic burden of the organs related to nitrogen metabolism was supposed to be decreased. However, different balanced patterns of amino acids in low protein diets had no effect on the organ index (*P* > 0.05), suggesting dietary CP level had a greater influence on the organ index than amino acid balance patterns.

### Nitrogen emission of growing-finishing pigs

Albumin (ALB) and globulin in serum reflect protein synthesis and nutritional status. Serum urea nitrogen (SUN) was an end product of the metabolism of proteins and amino acids, and its concentration was negative correlated with the utilization of proteins and amino acids [36]. NH_3_-N is a metabolite of intestinal microbial decomposition of protein and amino acids, which reflects the utilization of protein and amino acids. In this study, with balanced 10 EAAs, a 2% or 4% reduction in dietary protein significantly decreased the concentration of SUN (*P* <  0.05) but did not affect serum TP, ALB and NH_3_-N contents (Table 5). Similar results were found in Figueroa et al. [16], in which a 4% reduction of dietary protein significantly reduces the concentration of SUN in growing pigs. Furthermore, in low protein diet, addition of extra Ile significantly reduced the concentration of serum NH_3_-N (*P* <  0.05), compared with diet only balanced for Lys, Met, Thr and Trp. It was indicated that supplementation with Ile improved the amino acid balance in low protein diets and increased N utilization resulting in lower NH_3_-N levels. There was no significant difference in serum SUN, TP and ALB between the different amino acids addition diets, which was consistent with previous studies [18, 34]. Lordelo et al. [18] suggested that the changes in the concentration of serum amino acid and urea nitrogen had no aid in the identification of limiting amino acids in the diet.

**Table 5:** Blood profiles of finishing pigs fed low protein diet supplemented with different amino acids (*n* = 6)

Item	HCP	MCP (−2, %)	LCP(−4, %)					*P*_1_	*P*_2_
	10 EAAs	10 EAAs	10 EAAs	Lys, Met, Thr, Trp	Lys, Met,Thr, Trp, Ile	Lys, Met, Thr, Trp, Val	Lys, Met, Thr, Trp, Ile, Val		
SUN, mmol/L	7.35 ± 0.38^x^	4.62 ± 0.20^y^	3.98 ± 0.33^y^	4.74 ± 0.38	3.94 ± 0.28	4.15 ± 0.43	4.56 ± 0.55	< 0.01	0.51
ALB, g/L	40.78 ± 1.71	41.19 ± 2.15	43.89 ± 2.84	41.29 ± 2.73	41.26 ± 2.01	43.50 ± 2.75	41.1 ± 3.95	0.59	0.92
TP, g/L	57.92 ± 1.45	58.94 ± 1.19	60.11 ± 2.17	56.28 ± 1.88	57.35 ± 1.05	59.26 ± 2.16	62.56 ± 1.14	0.66	0.17
NH_3_-N, mg/L	40.64 ± 1.96	34.29 ± 3.05	35.09 ± 1.19^ab^	37.33 ± 1.46^a^	31.75 ± 0.77^b^	36.73 ± 1.41^a^	35.36 ± 1.32^ab^	0.13	0.01

SUN, Serum urea nitrogen; ALB, Albumin; TP, Total protein.

*P*_1_ represented the *P* value of ANOVA among the HCP diet, MCPdiet and LCP diet with balancing 10 EAAs.

*P*_2_ represented the *P* value of ANOVA among different amino acid balanced patterns of the LCP diets groups.

^x,y,z^Superscripts indicate differences among different levels of dietary protein

Reduce the dietary protein level and supplementation of crystalline amino acids is an effective way to reduce N excretion without affecting animal growth performance. In this study, when the dietary protein level was reduced by 2% or 4% and balancing 10 EAAs, total nitrogen excretion decreased by 16.9% and 31.9%, respectively (*P* <  0.05) (Table 6), which was consistent with previous studies [37, 38]. Also, the N intake and N excretion in 4% lower protein diet were 11.6% and 26.1% less than that of the 2% reduction diet, respectively. This result indicated that total N excretion could be reduced by 8.2% for every one percent reduction in dietary protein, consisting with the previous summary [35]. In 4% lower protein diet, the total N excretion in groups supplemented with only Lys, Met, Thr and Trp was numerically higher than that of other groups. Specifically, in the 25–50 kg phase, the total N excretion was 29.5% higher than that of 10 EAAs balanced diet and 22.0% higher than that of Ile alone addition diet. In summary, both dietary protein levels and amino acid balance patterns would affect the utilization and excretion of N.

**Table 6:** Nitrogen balance of growing-finishing pigs fed low protein diet supplemented with different amino acids (*n* = 6)

Item	HCP	MCP (− 2, %)	LCP(− 4, %)					*P*_1_	*P*_2_
	10 EAAs	10 EAAs	10 EAAs	Lys, Met,Thr, Trp	Lys, Met,Thr, Trp, Ile	Lys, Met,Thr, Trp, Val	Lys, Met,Thr, Trp, Ile, Val		
**PhaseI (25–50, kg)**									
N intake, g/d	53.00 ± 3.00^x^	48.23 ± 2.25^x^	40.72 ± 0.62^yab^	40.42 ± 3.06^ab^	34.40 ± 2.43^b^	42.97 ± 2.64^a^	41.04 ± 1.16^ab^	< 0.01	0.08
FN, g/d	6.56 ± 0.45^x^	6.32 ± 0.42^x^	4.79 ± 0.57^y^	5.67 ± 0.57	4.50 ± 0.42	5.92 ± 0.64	5.32 ± 0.11	0.04	0.27
UN, g/d	10.12 ± 1.44^x^	7.28 ± 0.92^x^	4.04 ± 0.52^y^	6.87 ± 0.90	5.26 ± 1.20	4.82 ± 0.84	5.40 ± 0.88	< 0.01	0.28
T N, g/d	16.69 ± 1.35^x^	13.60 ± 1.20^x^	8.83 ± 0.62^yb^	12.52 ± 1.05^a^	9.76 ± 1.17^ab^	10.74 ± 0.90^ab^	10.72 ± 0.86^ab^	< 0.01	0.09
R N, g/d	36.32 ± 2.44	34.62 ± 1.54	31.88 ± 0.93^a^	27.90 ± 2.16^ab^	24.64 ± 1.81^b^	32.23 ± 3.02^a^	30.31 ± 1.59^ab^	0.23	0.73
N retention, %	68.42 ± 1.99^y^	71.94 ± 1.65^y^	78.26 ± 1.60^xa^	69.02 ± 0.96^b^	71.73 ± 2.26^ab^	74.23 ± 3.37^ab^	73.70 ± 2.35^ab^	< 0.01	0.89
N ABV, %	78.22 ± 2.51^y^	82.84 ± 1.76^xy^	88.73 ± 1.47^xa^	80.28 ± 1.77^b^	82.83 ± 3.10^ab^	86.69 ± 2.43^ab^	84.70 ± 2.61^ab^	< 0.01	0.13
**Phase II (50–75, kg)**									
N intake (g/d)	55.82 ± 2.32^x^	45.71 ± 4.21^y^	43.69 ± 0.58^ya^	41.45 ± 1.03^ab^	37.32 ± 2.96^b^	43.10 ± 1.48^a^	43.87 ± 1.93^a^	< 0.01	0.09
FN (g/d)	8.53 ± 1.18	7.07 ± 0.71	6.62 ± 0.38	7.17 ± 0.36	5.70 ± 0.60	7.04 ± 0.59	6.65 ± 0.64	0.26	0.34
UN (g/d)	11.53 ± 1.89^x^	7.56 ± 1.20^xy^	5.98 ± 0.59^y^	6.12 ± 0.72	6.23 ± 0.41	7.49 ± 1.23	7.28 ± 0.72	0.03	0.53
T N (g/d)	20.06 ± 2.71^x^	14.62 ± 1.57^xy^	12.59 ± 0.43^y^	13.29 ± 0.59	11.93 ± 0.61	14.53 ± 1.24	13.93 ± 1.23	0.03	0.20
R N (g/d)	35.75 ± 2.73	31.06 ± 3.41	31.10 ± 0.85	28.16 ± 1.00	25.39 ± 2.48	28.58 ± 2.51	29.95 ± 1.82	0.32	0.29
N retention rate (%)	64.21 ± 4.19	67.68 ± 2.46	71.12 ± 1.20	67.89 ± 1.34	69.53 ± 1.48	65.71 ± 3.97	68.16 ± 2.45	0.27	0.60
N ABV(%)	75.54 ± 4.02	80.13 ± 2.85	83.84 ± 1.64	82.19 ± 1.92	79.67 ± 1.93	78.57 ± 4.12	80.20 ± 2.13	0.17	0.60
**Phase III (75–100, kg)**									
N intake, g/d	67.57 ± 1.57^x^	55.85 ± 2.94^y^	49.04 ± 1.65^z^	45.51 ± 0.96	47.42 ± 3.05	47.18 ± 2.80	47.11 ± 3.77	< 0.01	0.92
FN, g/d	6.53 ± 0.37^x^	5.71 ± 0.52^xy^	5.12 ± 0.35^y^	5.43 ± 0.43	4.86 ± 0.45	4.50 ± 0.31	4.94 ± 0.37	0.07	0.54
UN, g/d	17.49 ± 3.58^x^	14.18 ± 1.0^xy^	9.62 ± 0.98^y^	10.82 ± 1.68	9.79 ± 1.04	10.71 ± 2.15	10.80 ± 1.83	0.08	0.96
T N, g/d	24.02 ± 3.80^x^	19.89 ± 1.10^xy^	14.74 ± 1.13^y^	16.25 ± 1.73	14.65 ± 1.39	15.20 ± 2.27	15.74 ± 2.05	0.05	0.96
R N, g/d	43.54 ± 2.69^x^	35.96 ± 2.13^y^	34.30 ± 1.47^y^	29.26 ± 1.54	32.76 ± 3.58	31.97 ± 2.00	31.37 ± 2.50	0.02	0.68
N retention, %	64.86 ± 4.73	64.33 ± 1.18	69.96 ± 1.92	64.40 ± 3.58	67.68 ± 5.06	68.22 ± 3.74	66.81 ± 2.81	0.41	0.86
N ABV, %	71.78 ± 5.60	71.67 ± 1.45	78.06 ± 1.90	73.13 ± 4.04	75.36 ± 4.73	75.43 ± 4.23	74.88 ± 3.03	0.35	0.92
**Phase IV (100–125, kg)**									
N intake, g/d	63.49 ± 2.62^x^	50.22 ± 1.71^y^	43.62 ± 1.40^z^	44.61 ± 1.66	44.24 ± 1.89	44.34 ± 1.85	40.38 ± 4.96	< 0.01	0.77
FN, g/d	5.21 ± 2.09	5.11 ± 2.70	4.14 ± 1.21	4.66 ± 2.02	4.89 ± 1.55	4.91 ± 2.42	4.16 ± 0.75	0.18	0.69
UN, g/d	25.59 ± 0.48^x^	15.71 ± 0.36^y^	10.08 ± 0.42^y^	11.14 ± 0.44	9.89 ± 0.54	10.71 ± 0.40	11.69 ± 0.71	< 0.01	0.95
T N, g/d	30.80 ± 2.15^x^	20.82 ± 3.02^y^	14.22 ± 1.09^z^	15.80 ± 1.93	14.77 ± 1.12	15.62 ± 2.67	15.85 ± 0.80	< 0.01	0.95
R N, g/d	32.69 ± 2.03	29.40 ± 4.46	29.40 ± 1.55	28.80 ± 2.42	29.46 ± 1.41	28.73 ± 3.23	24.52 ± 4.64	0.62	0.73
N retention, %	51.55 ± 2.65^y^	57.63 ± 7.82^xy^	67.33 ± 2.37^x^	64.40 ± 4.24	66.67 ± 2.01	64.53 ± 6.36	57.67 ± 6.55	0.07	0.64
N ABV, %	56.18 ± 3.06^y^	63.88 ± 8.19^xy^	74.44 ± 2.84^x^	71.98 ± 4.99	75.38 ± 3.55	72.35 ± 6.68	64.32 ± 7.62	0.05	0.65

F N, Fecal nitrogen; U N, Urine nitrogen; T N, Total nitrogen; R N, Retained nitrogen; ABV, Apparent biological value.

*P*_1_ represented the *P* value of ANOVA among the HCP diet, MCPdiet and LCP diet with balancing 10 EAAs.

*P*_2_ represented the *P* value of ANOVA among different amino acid balanced patterns of the LCP diets groups.

^x,y,z^Superscripts indicate differences among different levels of dietary protein

The nitrogen in urine is mainly in the form of urea. When mixed with faeces, urea can be quickly decomposed into carbon dioxide and ammonia by the urease in the faeces [39, 40]. Therefore, the volatilization of ammonia in pig excreta is closely related to the amount of nitrogen excretion. Several studies found that low-protein diets can reduce nitrogen excretion and ammonia emissions [41, 42]. In this study, 2% lower protein diet with balanced 10 EAAs significantly decreased slurry ammonia volatilization at 4 h in each phase(*P* <  0.05). 4% lower dietary protein diet significantly decreased slurry ammonia volatilization at 0 h and 4 h in each phase (*P* <  0.05) (Table 7). However, there was no significant difference in slurry ammonia volatilization at 0 h and 4 h between the different amino acids balanced patterns of low protein diets. It suggested that the volatilization of excrement ammonia was mainly affected by dietary protein level.

**Table 7:** Slurry NH_3_ emission of growing-finishing pigs fed low protein diet supplemented with different amino acids (*n* = 6)

Item	HCP	MCP (− 2, %)	LCP (− 4, %)					*P*_1_	*P*_2_
	10 EAAs	10 EAAs	10 EAAs	Lys, Met,Thr, Trp	Lys, Met,Thr, Trp, Ile	Lys, Met,Thr, Trp, Val	Lys, Met,Thr, Trp, Ile, Val		
**0 h, mg/m** ^**3**^									
Phase I 50, kg	95.06 ± 17.13^x^	65.05 ± 12.99^xy^	33.06 ± 9.06^y^	37.37 ± 7.13	29.43 ± 8.37	31.11 ± 13.76	37.36 ± 9.50	0.02	0.96
Phase II 75, kg	23.43 ± 3.60^x^	9.43 ± 1.99^y^	8.65 ± 1.05^y^	9.44 ± 1.15	9.86 ± 2.32	8.89 ± 2.33	9.25 ± 1.79	< 0.01	0.89
Phase III 100, kg	19.98 ± 4.39^x^	8.45 ± 1.12^y^	4.39 ± 0.61^y^	4.12 ± 0.36	4.83 ± 0.63	4.01 ± 1.79	2.85 ± 0.58	< 0.01	0.66
Phase IV 125, kg	30.48 ± 8.43^x^	15.26 ± 4.01^xy^	7.25 ± 2.10^y^	9.29 ± 1.82	5.51 ± 2.01	9.27 ± 1.39	10.76 ± 1.36	0.03	0.28
**4 h, mg/m** ^**3**^									
Phase I 50, kg	275.44 ± 23.37^x^	156.01 ± 26.53^y^	75.51 ± 16.85^z^	66.67 ± 9.44	66.53 ± 17.32	67.99 ± 15.99	78.09 ± 10.47	< 0.01	0.96
Phase II 75, kg	189.89 ± 28.81^x^	62.94 ± 14.14^y^	43.72 ± 5.79^y^	54.17 ± 11.87	54.99 ± 13.27	49.06 ± 7.82	54.86 ± 11.74	< 0.01	0.92
Phase III 100, kg	118.81 ± 18.67^x^	57.71 ± 6.59^y^	32.39 ± 5.49^y^	28.44 ± 4.08	28.13 ± 2.92	27.82 ± 4.64	33.25 ± 2.66	< 0.01	0.81
Phase IV 125, kg	154.60 ± 25.74^x^	78.74 ± 6.93^y^	42.71 ± 3.54^y^	50.55 ± 7.87	44.93 ± 5.82	42.69 ± 3.92	47.52 ± 7.04	< 0.01	0.86

*P*_1_ represented the *P* value of ANOVA among the HCP diet, MCPdiet and LCP diet with balancing 10 EAAs.

*P*_2_ represented the *P* value of ANOVA among different amino acid balanced patterns of the LCP diets groups.

^x,y,z^Superscripts indicate differences among different levels of dietary protein

## Conclusions

Taken together, 2% or 4% decreases in dietary protein levels with balanced 10 EAAs had no significant effects on growth performance and carcass characteristics but significantly reduced nitrogen excretion in pigs. In 4% lower protein diets, Val supplementation significantly increased body weight gain at 25-50 kg phase, while Ile supplementation at 75-100 kg phase and 100–125 kg phase significantly reduced the ratio of feed to gain. The total N excretion of pigs supplemented with only Lys, Met, Thr and Trp was numerically higher than that of pigs fed with extra Ile, or Val, or Ile + Val diets. These results indicated that in low protein diet, Val is more required than Ile in the early growing phage (25–50 kg), while Ile becomes more required in the late growing and finishing phage (75–125 kg).

## Additional file

Additional file 1: **Table S1.** Ingredient and chemical composition of experimental diets during phase I 25–50 kg (as fed basis). **Table S2.** Ingredient and chemical composition of experimental diets during phase II 50–75 kg (as fed basis). **Table S3.** Ingredient and chemical composition of experimental diets during phase III 75–100 kg (as fed basis). **Table S4.** Ingredient and chemical composition of experimental diets during phase IV 100–125 kg (as fed basis). (DOCX 41 kb)

## Data Availability

The data analyzed during the current study are available from the corresponding author on reasonable request.

## Abbreviations

AA: Amnio acids
ADFI: Average daiy feed intake
ADG: Average daiy gain
ALB: Albumin
CP: Crude protein
EAA: Essential amnio acids
F/G: Feed to gain ratio
NE: Net energy
NH_3_-N: Ammonia nitrogen
NRC: National research council
TP: Total protein
