# Supplementary material for: Effect of different dietary protein levels and amino acids supplementation patterns on growth performance, carcass characteristics and nitrogen excretion in growing-finishing pigs
Source: J Anim Sci Biotechnol. 2019 Sep 16;10:75. doi: 10.1186/s40104-019-0381-2 (PMC6745769; doi:10.1186/s40104-019-0381-2)
Supplement: Supplementary file 1 — Table S1. Ingredient and chemical composition of experimental diets during phase I 25–50 kg (as fed basis). Table S2. Ingredient and chemical composition of experimental diets during phase II 50–75 kg (as fed basis). Table S3. Ingredient and chemical composition of experimental diets during phase III 75–100 kg (as fed basis). Table S4. Ingredient and chemical composition of experimental diets during phase IV 100–125 kg (as fed basis). (DOCX 41 kb) [file 40104_2019_381_MOESM1_ESM.docx]

**Additional file 1: Table S1.** Ingredient and chemical composition of experimental diets during phase I 25-50 kg (as fed basis)

| Ingredients | HCP | MCP | LCP | | | | |
| --- | --- | --- | --- | --- | --- | --- | --- |
|  | 10  EAAs^1^ | 10  EAAs | 10  EAAs | Lys Met Thr Trp | Lys Met Thr Trp Ile | Lys Met  Thr Trp Val | Lys Met  Thr Trp  Ile Val |
| Cron | 67.02 | 73.10 | 80.77 | 79.48 | 79.81 | 79.95 | 80.28 |
| Soybean meal | 26.42 | 20.25 | 11.18 | 12.17 | 11.99 | 11.86 | 11.65 |
| Rapeseed meal | 2.00 | 2.00 | 2.00 | 2.00 | 2.00 | 2.00 | 2.00 |
| Wheat bran |  |  | 1.96 | 2.89 | 2.57 | 2.49 | 2.21 |
| Soybean oil | 2.00 | 1.55 |  |  |  |  |  |
| Limestone | 0.66 | 0.64 | 0.65 | 0.68 | 0.67 | 0.66 | 0.66 |
| Dicalcium phosphate | 0.87 | 0.97 | 1.05 | 1.00 | 1.01 | 1.02 | 1.03 |
| NaCl | 0.30 | 0.30 | 0.30 | 0.30 | 0.30 | 0.30 | 0.30 |
| L-Lys-HCl (78%) | 0.24 | 0.42 | 0.68 | 0.65 | 0.66 | 0.66 | 0.67 |
| DL-Met (99%) | 0.08 | 0.13 | 0.20 | 0.19 | 0.20 | 0.20 | 0.20 |
| Thr (98.5%) | 0.04 | 0.11 | 0.23 | 0.21 | 0.22 | 0.22 | 0.22 |
| Trp (98%) |  | 0.03 | 0.07 | 0.06 | 0.06 | 0.06 | 0.07 |
| Ile(99%) |  | 0.03 | 0.15 |  | 0.14 |  | 0.15 |
| Val (99%) |  | 0.10 | 0.21 |  |  | 0.20 | 0.20 |
| Phe(99%) |  |  | 0.10 |  |  |  |  |
| His (99%) |  |  | 0.07 |  |  |  |  |
| Mineral Complex^2^ | 0.20 | 0.20 | 0.20 | 0.20 | 0.20 | 0.20 | 0.20 |
| Choline Chloride | 0.15 | 0.15 | 0.15 | 0.15 | 0.15 | 0.15 | 0.15 |
| Vitamin Complex ^3^ | 0.03 | 0.03 | 0.03 | 0.03 | 0.03 | 0.03 | 0.03 |
| Total | 100.00 | 100.00 | 100.00 | 100.00 | 100.00 | 100.00 | 100.00 |
| Nutrient |  |  |  |  |  |  |  |
| CP^4^% | 17.42 | 15.76 | 13.54 | 13.28 | 13.54 | 13.50 | 13.29 |
| NE ^5^MJ kg^-1^ | 10.36 | 10.36 | 10.06 | 10.06 | 10.06 | 10.06 | 10.06 |
| CF% | 2.93 | 2.63 | 2.36 | 2.46 | 2.44 | 2.42 | 2.39 |
| Calcium % | 0.66 | 0.66 | 0.67 | 0.67 | 0.67 | 0.67 | 0.66 |
| Available phosphorous % | 0.33 | 0.35 | 0.36 | 0.36 | 0.36 | 0.36 | 0.36 |
| SIDAA% |  |  |  |  |  |  |  |
| Lys | 0.98 | 0.98 | 0.98 | 0.98 | 0.98 | 0.98 | 0.98 |
| Met+Cys | 0.55 | 0.55 | 0.55 | 0.55 | 0.55 | 0.55 | 0.55 |
| Thr | 0.59 | 0.59 | 0.59 | 0.59 | 0.59 | 0.59 | 0.59 |
| Trp | 0.17 | 0.17 | 0.17 | 0.17 | 0.17 | 0.17 | 0.17 |
| Ile | 0.56 | 0.51 | 0.51 | 0.37 | 0.51 | 0.37 | 0.51 |
| Val | 0.64 | 0.64 | 0.64 | 0.45 | 0.45 | 0.64 | 0.64 |
| Leu | 1.29 | 1.17 | 0.99 | 1.01 | 1.01 | 1.01 | 1.00 |
| Arg | 1.06 | 0.90 | 0.66 | 0.69 | 0.69 | 0.68 | 0.67 |
| His | 0.39 | 0.34 | 0.34 | 0.28 | 0.28 | 0.28 | 0.27 |
| Phe | 0.72 | 0.63 | 0.59 | 0.51 | 0.51 | 0.50 | 0.50 |

^1)^ Balance of 10 essential amino acids include Lys, Met, Thr, Trp, Val, Ile, Phe, His, Leu, Arg;

^2)^ Supplied the following per kg of diet: Fe (FeSO_4_∙H_2_O) 60mg; Mn (MnSO_4_) 2mg; Zn (MnSO_4_)

60mg; Cu (CuSO_4_∙5H_2_O) 4mg; I(KI) 0.14mg; Se (Na_2_SeO_3_) 0.2 mg;

^3)^ Supplied the following per kg of diet: vitamin A, 5,512 IU; vitamin D3, 2,200 IU; vitamin E, 30 IU; vitamin K3, 2.2 mg; vitamin B12, 27.6 μg; riboflavin, 4 mg; pantothenic acid, 14 mg; niacin, 30 mg; folic acid, 0.7mg; thiamin, 1.5 mg; pyridoxine, 3 mg; biotin, 44 μg

^4)^ Analytical value

^5)^According to China Feed Composition and Nutritional Value Table (25th edition, 2014)

**Additional file 1: Table S2.** Ingredient and chemical composition of experimental diets during phase II 50-75 kg (as fed basis)

| Ingredients | HCP | MCP | LCP | | | | |
| --- | --- | --- | --- | --- | --- | --- | --- |
|  | 10  EAAs^1^ | 10  EAAs | 10  EAAs | Lys Met Thr Trp | Lys Met  Thr Trp Ile | Lys Met  Thr Trp Val | Lys Met  Thr Trp  Ile Val |
| Cron | 71.99 | 79.58 | 83.01 | 81.85 | 82.16 | 82.24 | 82.57 |
| Soybean meal | 21.24 | 13.57 | 5.4 | 6.27 | 6.06 | 6.01 | 5.8 |
| Rapeseed meal | 3 | 3 | 3 | 3 | 3 | 3 | 3 |
| Wheat bran |  |  | 4.82 | 5.69 | 5.41 | 5.36 | 5.06 |
| Soybean oil | 1.48 | 0.91 |  |  |  |  |  |
| Limestone | 0.6 | 0.57 | 0.64 | 0.66 | 0.65 | 0.66 | 0.65 |
| Dicalcium phosphate | 0.74 | 0.86 | 0.84 | 0.8 | 0.81 | 0.81 | 0.83 |
| NaCl | 0.3 | 0.3 | 0.3 | 0.3 | 0.3 | 0.3 | 0.3 |
| L-Lys-HCl (78%) | 0.21 | 0.44 | 0.67 | 0.63 | 0.64 | 0.64 | 0.65 |
| DL-Met (99%) | 0.04 | 0.11 | 0.17 | 0.16 | 0.16 | 0.16 | 0.16 |
| Thr (98.5%) | 0.02 | 0.12 | 0.22 | 0.2 | 0.21 | 0.21 | 0.21 |
| Trp (98%) |  | 0.03 | 0.07 | 0.06 | 0.07 | 0.07 | 0.07 |
| Ile(99%) |  | 0.05 | 0.16 |  | 0.15 |  | 0.16 |
| Val (99%) |  | 0.08 | 0.18 |  |  | 0.17 | 0.17 |
| Phe(99%) |  |  | 0.1 |  |  |  |  |
| His (99%) |  |  | 0.06 |  |  |  |  |
| Mineral Complex^2^ | 0.2 | 0.2 | 0.2 | 0.2 | 0.2 | 0.2 | 0.2 |
| Choline Chloride | 0.15 | 0.15 | 0.15 | 0.15 | 0.15 | 0.15 | 0.15 |
| Vitamin Complex ^3^ | 0.03 | 0.03 | 0.03 | 0.03 | 0.03 | 0.03 | 0.03 |
| Total | 100 | 100 | 100 | 100 | 100 | 100 | 100 |
| Nutrient |  |  |  |  |  |  |  |
| CP^4^% | 16.01 | 13.70 | 12.24 | 12.06 | 11.83 | 12.19 | 12.05 |
| NE ^5^MJ kg^-1^ | 14.1 | 13.87 | 13.31 | 13.35 | 13.34 | 13.34 | 13.33 |
| CF% | 10.36 | 10.36 | 10.06 | 10.06 | 10.06 | 10.06 | 10.06 |
| Calcium % | 2.76 | 2.43 | 2.33 | 2.42 | 2.39 | 2.39 | 2.36 |
| Available phosphorous % | 0.59 | 0.6 | 0.59 | 0.59 | 0.59 | 0.6 | 0.6 |
| SIDAA% | 0.31 | 0.31 | 0.31 | 0.31 | 0.31 | 0.31 | 0.31 |
| Lys |  |  |  |  |  |  |  |
| Met+Cys | 0.85 | 0.85 | 0.85 | 0.85 | 0.85 | 0.85 | 0.85 |
| Thr | 0.26 | 0.29 | 0.32 | 0.32 | 0.32 | 0.32 | 0.32 |
| Trp | 0.48 | 0.48 | 0.48 | 0.48 | 0.48 | 0.48 | 0.48 |
| Ile | 0.52 | 0.52 | 0.52 | 0.52 | 0.52 | 0.52 | 0.52 |
| Val | 0.15 | 0.15 | 0.15 | 0.15 | 0.15 | 0.15 | 0.15 |
| Leu | 0.5 | 0.45 | 0.45 | 0.31 | 0.45 | 0.3 | 0.45 |
| Arg | 0.57 | 0.55 | 0.55 | 0.39 | 0.39 | 0.55 | 0.55 |
| His | 1.2 | 1.05 | 0.88 | 0.9 | 0.9 | 0.9 | 0.89 |
| Phe | 0.93 | 0.72 | 0.54 | 0.57 | 0.56 | 0.56 | 0.55 |

^1)^ Balance of 10 essential amino acids include Lys, Met, Thr, Trp, Val, Ile, Phe, His, Leu, Arg

^2)^ Supplied the following per kg of diet: Fe (FeSO_4_∙H_2_O) 50mg; Mn (MnSO_4_) 2mg; Zn (MnSO_4_) 50mg; Cu (CuSO_4_∙5H_2_O) 3.5mg; I (KI) 0.14mg; Se (Na_2_SeO_3_) 0.15 mg;

^3)^ Supplied the following per kg of diet: vitamin A, 5,512 IU; vitamin D3, 2,200 IU; vitamin E, 30 IU; vitamin K3, 2.2 mg; vitamin B12, 27.6 μg; riboflavin, 4 mg; pantothenic acid, 14 mg; niacin, 30 mg; folic acid, 0.7mg; thiamin, 1.5 mg; pyridoxine, 3 mg; biotin, 44 μg

^4)^ Analytical value

^5)^According to China Feed Composition and Nutritional Value Table (25th edition, 2014)

**Additional file 1: Table S3.** Ingredient and chemical composition of experimental diets during phase III 75-100 kg (as fed basis)

| Ingredients | HCP | MCP | LCP | | | | |
| --- | --- | --- | --- | --- | --- | --- | --- |
|  | 10  EAAs^1^ | 10  EAAs | 10  EAAs | Lys Met Thr Trp | LysMetThr Trp Ile | LysMetThr Trp Val | Lys Met  Thr Trp  Ile Val |
| Cron | 79.20 | 84.73 | 85.62 | 84.26 | 84.56 | 84.59 | 84.97 |
| Soybean meal | 18.21 | 8.05 |  | 0.53 | 0.3 | 0.27 | 0.07 |
| Rapeseed meal |  | 4 | 4 | 4 | 4 | 4 | 4 |
| Wheat bran | 0.39 |  | 6.52 | 8.15 | 7.9 | 7.91 | 7.54 |
| Soybean oil | 0.55 | 0.41 |  |  |  |  |  |
| Limestone | 0.64 | 0.54 | 0.64 | 0.69 | 0.68 | 0.68 | 0.67 |
| Dicalcium phosphate | 0.4 | 0.68 | 0.63 | 0.56 | 0.57 | 0.57 | 0.59 |
| NaCl | 0.16 | 0.4 | 0.4 | 0.4 | 0.4 | 0.4 | 0.4 |
| L-Lys-HCl (78%) | 0.04 | 0.44 | 0.65 | 0.63 | 0.64 | 0.64 | 0.65 |
| DL-Met (99%) | 0.03 | 0.08 | 0.14 | 0.13 | 0.13 | 0.13 | 0.14 |
| Thr (98.5%) | 0 | 0.12 | 0.21 | 0.2 | 0.21 | 0.21 | 0.21 |
| Trp (98%) |  | 0.04 | 0.07 | 0.07 | 0.07 | 0.07 | 0.07 |
| Ile(99%) |  | 0.06 | 0.17 |  | 0.16 |  | 0.16 |
| Val (99%) |  | 0.07 | 0.16 |  |  | 0.15 | 0.16 |
| Phe(99%) |  |  | 0.1 |  |  |  |  |
| His (99%) |  |  | 0.05 |  |  |  |  |
| Mineral Complex^2^ | 0.20 | 0.20 | 0.2 | 0.20 | 0.20 | 0.20 | 0.20 |
| Choline Chloride | 0.15 | 0.15 | 0.15 | 0.15 | 0.15 | 0.15 | 0.15 |
| Vitamin Complex ^3^ | 0.03 | 0.03 | 0.03 | 0.03 | 0.03 | 0.03 | 0.03 |
| Ball milling chaff |  |  | 0.26 |  |  |  |  |
| Total | 100 | 100 | 100 | 100 | 100 | 100 | 100 |
| Nutrient |  |  |  |  |  |  |  |
| CP^4^% | 14.63 | 12.4 | 10.7 | 10.48 | 10.41 | 10.41 | 10.54 |
| NE ^5^MJ kg^-1^ | 10.36 | 10.36 | 10.06 | 10.06 | 10.06 | 10.06 | 10.06 |
| CF% | 2.60 | 2.41 | 2.32 | 2.41 | 2.38 | 2.38 | 2.36 |
| Calcium % | 0.52 | 0.52 | 0.52 | 0.52 | 0.52 | 0.52 | 0.53 |
| Available phosphorous % | 0.26 | 0.27 | 0.27 | 0.26 | 0.26 | 0.26 | 0.26 |
| SIDAA% |  |  |  |  |  |  |  |
| Lys | 0.73 | 0.73 | 0.73 | 0.73 | 0.73 | 0.73 | 0.73 |
| Met+Cys | 0.42 | 0.42 | 0.42 | 0.42 | 0.42 | 0.42 | 0.42 |
| Thr | 0.46 | 0.46 | 0.46 | 0.46 | 0.46 | 0.46 | 0.46 |
| Trp | 0.13 | 0.13 | 0.13 | 0.13 | 0.13 | 0.13 | 0.13 |
| Ile | 0.44 | 0.33 | 0.39 | 0.24 | 0.39 | 0.24 | 0.39 |
| Val | 0.52 | 0.41 | 0.48 | 0.34 | 0.33 | 0.48 | 0.48 |
| Leu | 1.12 | 0.94 | 0.79 | 0.81 | 0.8 | 0.8 | 0.79 |
| Arg | 0.82 | 0.59 | 0.43 | 0.46 | 0.45 | 0.45 | 0.44 |
| His | 0.32 | 0.25 | 0.25 | 0.21 | 0.21 | 0.21 | 0.21 |
| Phe | 0.59 | 0.45 | 0.44 | 0.36 | 0.36 | 0.36 | 0.35 |

^1)^ Balance of 10 essential amino acids include Lys, Met, Thr, Trp, Val, Ile, Phe, His, Leu, Arg

^2)^ Supplied the following per kg of diet: Fe (FeSO_4_∙H_2_O) 40mg; Mn (MnSO_4_) 2mg; Zn (MnSO_4_) 40mg; Cu (CuSO_4_∙5H_2_O) 3.5mg; I (KI) 0.14mg; Se (Na_2_SeO_3_) 0.15 mg

^3)^ Supplied the following per kg of diet: vitamin A, 5,512 IU; vitamin D3, 2,200 IU; vitamin E, 30 IU; vitamin K3, 2.2 mg; vitamin B12, 27.6 μg; riboflavin, 4 mg; pantothenic acid, 14 mg; niacin, 30 mg; folic acid, 0.7mg; thiamin, 1.5 mg; pyridoxine, 3 mg; biotin, 44 μg

^4)^ Analytical value

^5)^ According to China Feed Composition and Nutritional Value Table (25th edition, 2014)

**Additional file 1: Table S4.** Ingredient and chemical composition of experimental diets during phase Ⅳ 100-125 kg (as fed basis)

| Ingredients | HCP | MCP | LCP | | | | |
| --- | --- | --- | --- | --- | --- | --- | --- |
|  | 10  EAAs^1^ | 10  EAAs | 10  EAAs | Lys Met Thr Trp | LysMet  Thr Trp Ile | LysMetThr Trp Val | LysMet  Thr Trp  Ile Val |
| Cron | 82.21 | 84.76 | 87.67 | 86.35 | 86.77 | 86.8 | 87.23 |
| Soybean meal | 10.34 |  |  |  |  |  |  |
| Rapeseed meal | 5 | 5 |  |  |  |  |  |
| Wheat bran |  | 7.03 | 6.85 | 9.15 | 8.42 | 8.38 | 7.61 |
| Soybean oil | 0.48 |  |  |  |  |  |  |
| Limestone | 0.54 | 0.65 | 0.57 | 0.64 | 0.62 | 0.61 | 0.59 |
| Dicalcium phosphate | 0.43 | 0.4 | 0.59 | 0.51 | 0.54 | 0.54 | 0.56 |
| NaCl | 0.4 | 0.4 | 0.4 | 0.4 | 0.4 | 0.4 | 0.4 |
| L-Lys-HCl (78%) | 0.2 | 0.48 | 0.54 | 0.53 | 0.53 | 0.53 | 0.54 |
| DL-Met (99%) |  | 0.07 | 0.12 | 0.11 | 0.12 | 0.12 | 0.12 |
| Thr (98.5%) | 0.02 | 0.14 | 0.19 | 0.18 | 0.19 | 0.19 | 0.19 |
| Trp (98%) |  | 0.05 | 0.06 | 0.06 | 0.06 | 0.06 | 0.06 |
| Ile(99%) |  | 0.09 | 0.14 |  | 0.14 | 0 | 0.14 |
| Val (99%) |  | 0.08 | 0.14 |  |  | 0.13 | 0.13 |
| Phe(99%) |  |  | 0.07 |  |  |  |  |
| His (99%) |  |  | 0.03 |  |  |  |  |
| Mineral Complex^2^ | 0.2 | 0.2 | 0.2 | 0.2 | 0.2 | 0.2 | 0.2 |
| Choline Chloride | 0.15 | 0.15 | 0.15 | 0.15 | 0.15 | 0.15 | 0.15 |
| Vitamin Complex ^3^ | 0.03 | 0.03 | 0.03 | 0.03 | 0.03 | 0.03 | 0.03 |
| Ball milling chaff |  | 0.48 | 2.25 | 1.69 | 1.85 | 1.87 | 2.05 |
| Total | 100 | 100 | 100 | 100 | 100 | 100 | 100 |
| Nutrient |  |  |  |  |  |  |  |
| CP^4^% | 13.11 | 10.67 | 9.33 | 9.12 | 9.21 | 9.21 | 9.18 |
| NE ^5^MJ kg^-1^ | 10.36 | 10.06 | 10.06 | 10.06 | 10.06 | 10.06 | 10.06 |
| CF% | 2.52 | 2.42 | 1.87 | 2 | 1.96 | 1.96 | 1.91 |
| Calcium % | 0.46 | 0.46 | 0.47 | 0.47 | 0.47 | 0.47 | 0.46 |
| Available phosphorous % | 0.23 | 0.22 | 0.25 | 0.24 | 0.24 | 0.24 | 0.25 |
| SIDAA% |  |  |  |  |  |  |  |
| Lys | 0.61 | 0.61 | 0.61 | 0.61 | 0.61 | 0.61 | 0.61 |
| Met+Cys | 0.37 | 0.36 | 0.36 | 0.36 | 0.36 | 0.36 | 0.36 |
| Thr | 0.4 | 0.4 | 0.4 | 0.4 | 0.4 | 0.4 | 0.4 |
| Trp | 0.11 | 0.11 | 0.11 | 0.11 | 0.11 | 0.11 | 0.11 |
| Ile | 0.37 | 0.33 | 0.33 | 0.2 | 0.33 | 0.2 | 0.33 |
| Val | 0.45 | 0.41 | 0.41 | 0.29 | 0.28 | 0.41 | 0.41 |
| Leu | 1.01 | 0.8 | 0.73 | 0.74 | 0.73 | 0.73 | 0.73 |
| Arg | 0.66 | 0.43 | 0.36 | 0.38 | 0.38 | 0.38 | 0.37 |
| His | 0.28 | 0.21 | 0.21 | 0.18 | 0.18 | 0.18 | 0.18 |
| Phe | 0.5 | 0.35 | 0.37 | 0.32 | 0.31 | 0.31 | 0.31 |

^1)^ Balance of 10 essential amino acids include Lys, Met, Thr, Trp, Val, Ile, Phe, His, Leu, Arg

^2)^ Supplied the following per kg of diet: Fe (FeSO_4_∙H_2_O) 40mg; Mn (MnSO_4_) 2mg; Zn (MnSO_4_) 40mg; Cu (CuSO_4_∙5H_2_O) 3.5mg; I(KI) 0.14mg; Se (Na_2_SeO_3_) 0.15 mg

^3)^ Supplied the following per kg of diet: vitamin A, 5,512 IU; vitamin D3, 2,200 IU; vitamin E, 30 IU; vitamin K3, 2.2 mg; vitamin B12, 27.6 μg; riboflavin, 4 mg; pantothenic acid, 14 mg; niacin, 30 mg; folic acid, 0.7mg; thiamin, 1.5 mg; pyridoxine, 3 mg; biotin, 44 μg

^4)^ Analytical value

^5)^ According to China Feed Composition and Nutritional Value Table (25th edition, 2014)
